# Magnolol Attenuates Right Ventricular Hypertrophy and Fibrosis in Hypoxia-Induced Pulmonary Arterial Hypertensive Rats Through Inhibition of the JAK2/STAT3 Signaling Pathway

**DOI:** 10.3389/fphar.2021.755077

**Published:** 2021-10-26

**Authors:** Minyi Fu, Fangmei Luo, Eli Wang, Yueping Jiang, Shao Liu, Jun Peng, Bin Liu

**Affiliations:** ^1^ Department of Pharmacy, Xiangya Hospital, Central South University, Changsha, China; ^2^ National Clinical Research Center for Geriatric Disorders, Xiangya Hospital, Institute for Rational and Safe Medication Practices, Central South University, Changsha, China; ^3^ The Hunan Institute of Pharmacy Practice and Clinical Research, Xiangya Hospital, Central South University, Changsha, China; ^4^ Department of Pharmacy, Hunan Children’s Hospital, Changsha, China; ^5^ Department of Pharmacology, Xiangya School of Pharmaceutical Sciences, Central South University, Changsha, China; ^6^ Hunan Provincial Key Laboratory of Cardiovascular Research, Xiangya School of Pharmaceutical Sciences, Central South University, Changsha, China

**Keywords:** magnolol, right ventricle remodeling, myocardial hypertrophy, myocardial fibrosis, JAK2, stat3

## Abstract

Right ventricular (RV) remodeling is one of the essential pathological features in pulmonary arterial hypertension (PAH). RV hypertrophy or fibrosis are the leading causes of RV remodeling. Magnolol (6, 6′, 7, 12-tetramethoxy-2,2′-dimethyl-1-β-berbaman, C18H18O2) is a compound isolated from *Magnolia Officinalis*. It possesses multiple pharmacological activities, such as anti-oxidation and anti-inflammation. This study aims to evaluate the effects and underlying mechanisms of magnolol on RV remodeling in hypoxia-induced PAH. *In vivo*, male Sprague Dawley rats were exposed to 10% O_2_ for 4 weeks to establish an RV remodeling model, which showed hypertrophic and fibrotic features (increases of Fulton index, cellular size, hypertrophic and fibrotic marker expression), accompanied by an elevation in phosphorylation levels of JAK2 and STAT3; these changes were attenuated by treating with magnolol. *In vitro*, the cultured H9c2 cells or cardiac fibroblasts were exposed to 3% O_2_ for 48 h to induce hypertrophy or fibrosis, which showed hypertrophic (increases in cellular size as well as the expression of ANP and BNP) or fibrotic features (increases in the expression of collagen Ⅰ, collagen Ⅲ, and α-SMA). Administration of magnolol and TG-101348 or JSI-124 (both JAK2 selective inhibitors) could prevent myocardial hypertrophy and fibrosis, accompanied by the decrease in the phosphorylation level of JAK2 and STAT3. Based on these observations, we conclude that magnolol can attenuate RV hypertrophy and fibrosis in hypoxia-induced PAH rats through a mechanism involving inhibition of the JAK2/STAT3 signaling pathway. Magnolol may possess the potential clinical value for PAH therapy.

## Introduction

Pulmonary arterial hypertension (PAH) is a malignant cardiopulmonary vascular disease characterized by a progressive increase in pulmonary vascular resistance and pulmonary arterial pressure, which eventually leads to right ventricular (RV) remodeling and even RV failure (Vonk-Noordegraaf et al., 2013; Zelt et al., 2019). In PAH, the continuous increase in RV afterload can initially lead to compensatory remodeling of the right ventricle. However, as the disease progresses, it will gradually develop into decompensated remodeling, manifesting as excessive myocardial hypertrophy and fibrosis (Vonk Noordegraaf et al., 2017; Andersen et al., 2019; de Man et al., 2019). A cohort study has verified that the 5-year survival rate for PAH patients with stable or improving RV function is significantly higher than that of patients with RV failure (van de Veerdonk et al., 2011), suggesting that RV failure is the leading cause for the death in PAH patients. Current clinical drug treatment strategies for RV failure aim to enhance RV contractility or reduce RV afterload (Cassady and Ramani, 2020). However, it cannot effectively reverse the process of RV remodeling and RV failure during PAH. Therefore, seeking drugs with the potential to target the RV remodeling during PAH is of great significance for delaying the progression of PAH and improving the survival rate of PAH patients.

Janus kinase (JAK)/signal transducer and activator of transcription (STAT) is a classic membrane-to-nucleus signaling pathway that can be activated by diverse cytokines, growth factors, and interferons (O'Shea et al., 2015). In mammals, there are 4 JAKs (JAK1, JAK2, JAK3, and TYK2) and 7 STATs (STAT1, STAT2, STAT3, STAT4, STAT5A, STAT5B, and STAT6) (Villarino et al., 2020). Recently studies showed that the JAK2/STAT3 pathway is involved in the development of PAH (Milara et al., 2018; Zhang et al., 2020; Yerabolu et al., 2021). Zhang et al. confirmed that hypoxia could stimulate the phosphorylation of JAK2, which in turn activates STAT3. The activated STAT3 enters the nucleus to regulate the gene expression of *CyclinA2*, which ultimately leads to the excessive proliferation of pulmonary artery smooth muscle cells and participates in vascular remodeling of PAH (Zhang et al., 2020). Furthermore, Ye et al. demonstrated that the JAK2/STAT3 pathway participates in myocardial hypertrophy and fibrosis induced by angiotensin Ⅱ (AngⅡ) by regulating the expression of downstream target genes (such as *Tgf-β*, *Col1a1*, and *Myh7*), and ultimately lead to cardiac remodeling (Ye et al., 2020). Based on these reports, we hypothesized that the JAK2/STAT3 pathway might be a valuable strategy to prevent the development of RV remodeling in PAH.

Magnolol, a compound isolated from *Magnolia Officinalis*, possesses multiple pharmacological activities such as anti-oxidation, anti-inflammation, and anti-tumor (Zhang et al., 2019; Lin et al., 2021). A recent study has found that magnolol can inhibit the proliferation and collagen synthesis of cardiac fibroblasts (Chen et al., 2021b). In another report, magnolol can inhibit the phosphorylation of STAT3 in a dose-dependent manner, but its regulatory effect on JAK2 remains unclear (Peng et al., 2021). By using the SwissTargetPrediction database, we found that JAK2 may be a potential target of magnolol. Based on these reports and our prediction, it is reasonable to speculate that magnolol can prevent the development of RV remodeling during PAH through mechanisms involving blocking the activation of the JAK2/STAT3 pathway.

The main purpose of this study is to explore the effect of magnolol on RV hypertrophy and fibrosis in hypoxia-induced PAH rats. Using a rat model of hypoxia-induced PAH, we first investigated the beneficial effect of magnolol on RV remodeling and its relevance to the JAK2/STAT3 pathway. To confirm the findings *in vivo*, we established hypoxia-induced cell hypertrophy and fibrosis models by using H9c2 cells and cardiac fibroblasts, respectively. Combining with TG-101348 and JSI-124, the specific inhibitor of JAK2, we confirmed that the inhibitory effect of magnolol on myocardial hypertrophy and fibrosis is related to the inhibition of the JAK2/STAT3 signaling pathway.

## Materials and Methods

### Animal Experiments

Male SD rats (220 g) were randomly divided into five groups (*n* = 10 per group): the normoxia group, the hypoxia group, the hypoxia plus Magnolol (L) group (low dose, 10 mg/kg/d), the hypoxia plus Magnolol (H) group (high dose, 20 mg/kg/d), and the vehicle group. Rats in the normoxia group were kept in a normoxia environment for 4 weeks, while rats in the hypoxia group were kept in a hypoxic chamber (10% O_2_). Magnolol (purity ≥98%) was purchased from Energy Chemical Company. The rats in the hypoxia plus magnolol groups were administered with magnolol at 10 or 20 mg/kg (i.p.) once a day for 4 weeks. The rats in the hypoxia plus vehicle group were given the same volume (0.1 ml/100 g per day) of vehicle (a mixture with 5% DMSO, 30% PEG 400, 5% Tween-80, and 60% normal saline) and then subjected to hypoxia. At the end of 4 weeks, the heart function was assessed by Doppler echocardiography. And then, the rats were anesthetized with sodium pentobarbital (30 mg/kg, i.p.). The RVSP was measured by the right heart catheterization method. The heart tissues were collected and dissected to calculate the Fulton index (RV/LV + IVS, RV/tibial length, or RV/body weight). Part of the RV samples was fixed with 4% paraformaldehyde for morphological analysis, while other samples were frozen at −80°C for molecular studies (measurements of ANP, BNP, α-SMA, and collagen Ⅰ/Ⅲ mRNA expression as well as p-JAK2/JAK2 and p-STAT3/STAT3 protein levels).

### Cell Experiments

The rat heart-derived H9c2 cells were obtained from the Chinese Academy of Sciences (Shanghai, China). H9c2 cells were cultured in Dulbecco’s Modified Eagle Medium (DMEM) containing 10% fetal bovine serum (FBS). The isolation and culture of cardiac fibroblasts from the heart tissues of neonatal male rats were performed as in previous studies (Jeppesen et al., 2011; Li et al., 2016; Li et al., 2020). Briefly, the heart tissues of neonatal rats were harvested and cut into 1 mm^2^ pieces with scissors, which were digested in an incubator at 37°C for 20 min with trypsin/EDTA (Gibco, USA) and collagenase Ⅱ (Sigma Aldrich, USA). The supernatant was collected and centrifuged at 1,200 rpm for 8 min. Then the adherent cells were resuspended with DMEM containing 10% FBS and plated on the culture flask for 1.5 h. The identification of cardiac fibroblasts was performed by immunofluorescence staining (vimentin and α-SMA) as described in previous studies (Li et al., 2016; Li et al., 2020). Cells that are positive for vimentin and negative for α-SMA are cardiac fibroblasts. These cells were collected for subsequent experiments at the passage from 2 to 3.

To evaluate the effect of magnolol on hypoxia-induced myocardial hypertrophy and fibrosis, H9c2 or cardiac fibroblasts were divided into seven groups: 1) the control group, cells were cultured under normal conditions; 2) the hypoxia group, cells were cultured under hypoxic condition (3% O_2_); 3) the hypoxia plus magnolol group (L), 10 μM of magnolol was added to the culture medium before the hypoxia treatment; 4) the hypoxia plus magnolol group (H), 20 μM of magnolol was added to the culture medium before the hypoxia treatment; 5) the hypoxia plus TG-101348 group, 1 μM of TG-101348 (a specific inhibitor of JAK2) was added to the culture medium before the hypoxia treatment; 6) the hypoxia plus JSI-124 group, 1 μM of JSI-124 (a specific inhibitor of JAK2) was added to the culture medium before the hypoxia treatment; and 7) the hypoxia plus vehicle group, an equal volume of vehicle (DMSO) was added to the culture medium before the hypoxia treatment. At the end of the experiments, the cells were collected for morphological and molecular analysis.

### Echocardiographic Assessment

The echocardiographic assessment was conducted using a Vevo 2100 imaging system (Visual Sonics, Toronto, Canada) to evaluate the changes in RV function in rats. The RV wall thickness in the diastole and systole period was measured by short axis in motion mode. The differences in the ratio of pulmonary artery acceleration time to ejection time (PAAT/PAET) were calculated to evaluate the RV function in rats.

### Morphological Observation

The staining with Hematoxylin-eosin (HE), Wheat germ agglutinin (WGA), Sirius red, Masson’ trichrome (Masson), or Verhoeff elastic van Gieson (EVG) was performed to evaluate the morphological changes of the RV tissues. The procedures were conducted as described in our previous studies (Liu et al., 2014; Li et al., 2019a; Wang et al., 2019). Briefly, for HE staining, the paraffin sections were stained with HE staining solution (Servicebio, Wuhan, China) for 5 min. A minimum of 6 microscopic fields from each slide was randomly selected for observation under a microscope (Nikon, Tokyo, Japan). The cross-sectional width of RV tissue in each group was randomly measured to assess the degree of RV hypertrophy. 3 representative points per RV were chosen to measure the width, and the average value was used to represent the width for each RV to ensure the reliability of the results.

For WGA staining, the slices were deparaffinized and immersed in EDTA buffer for antigen retrieval. After washing 3 times with PBS, the slices were incubated with WGA staining solution (Servicebio, Wuhan, China) in the dark at 37°C for 30 min. DAPI staining solution was added to stain the nucleus for 5 min. The cross-sectional area and perimeter of 10 cells in the field of view were measured to obtain the average value. The Changes in the cross-sectional area and circumference of cardiomyocytes in the RV tissues were observed under a fluorescence microscope to assess the degree of myocardial hypertrophy.

For EVG staining, the paraffin sections of heart tissue were deparaffinized and stained with EVG staining solution (Servicebio, Wuhan, China) for 5 min, differentiated in 2% ferric chloride solution, and washed in running tap water. The formation of fibers in heart tissue was observed and analyzed under a microscope. The elastic fibers are purple-black, the collagen fibers are red, and the background is yellow.

For Sirius red or Masson staining, the slices were deparaffinized and stained with Sirius Red or Masson staining solution (Servicebio, Wuhan, China), the formation of collagen fibers in the interstitial or perivascular of RV tissue was observed under a microscope.

### Immunofluorescence Staining

Morphological changes in H9c2 cells were observed by immunofluorescence staining as described in our previous studies (Li et al., 2019b). In brief, H9c2 cells were fixed with 4% paraformaldehyde for 20 min. After washing with PBS 3 times, the cells were permeabilized with 0.25% Triton-X-100 (Beyotime, Shanghai, China) and blocked with bovine serum albumin (Merck, Darmstadt, Germany) for 45 min. Then the cells were incubated with primary antibodies against α-SMA (Cell Signaling Technology, Massachusetts, USA) overnight at 4°C followed by incubation with the secondary antibody of Alexa Fluor 488-labeled Goat Anti-Mouse IgG (Beyotime, Shanghai, China). The cell morphological changes were observed under a fluorescence microscope, and the cross-sectional area of H9c2 cells was calculated to assess the degree of cell hypertrophy.

Tissue immunofluorescence staining was performed to evaluate the phosphorylation level of JAK2 in the RV tissue. After the treatment of deparaffinization, antigen retrieval, and blocking, RV tissue slices were incubated with primary antibodies against p-JAK2 (Abcam, Cambridge, MA, USA) overnight at 4°C followed by incubation with the secondary antibody of Cy3-labeled Goat Anti-Rabbit IgG (Beyotime, Shanghai, China). After washing with PBS 3 times, the cell nucleus was incubated with DAPI solution at room temperature for 5 min. The intensity of red fluorescence under a fluorescence microscope was observed to evaluate the level of p-JAK2 in RV tissues.

### Real-Time PCR

Real-time PCR was performed to detect the mRNA level of ANP, BNP, collagen Ⅰ, collagen Ⅲ, and α-SMA. The real-time PCR primers for ANP, BNP, collagen Ⅰ, collagen Ⅲ, α-SMA, and GAPDH are displayed in Table 1. Briefly, total RNA was isolated and extracted from RV tissues, H9c2 cells, or cardiac fibroblasts according to the RNAiso Plus kit instructions (TaKaRa Biomedical Technology, Beijing, China). 500 ng of RNA from each sample was subjected to reverse transcription reaction according to the PrimeScript™ RT Master Mix kit instructions (TaKaRa Biomedical Technology, Beijing, China). Then, a 20 μl real-time PCR reaction mixture containing 4 μl cDNA template, 10 μl PerfectStart™ Green qPCR SuperMix (Transgen Biotech, Beijing, China), 0.4 μl Passive Reference Dye, 4.8 μl Nuclease-free water, and 0.4 μl of each primer was amplified according to the following steps: an initial predenaturation at 94°C for 30 s, followed by 40 cycles of PCR reaction at 94°C for 5 s, annealing and extension at 60°C for 31 s. Gene expression was quantified using GAPDH as a loading control.

**TABLE 1:** Primers for real-time PCR.

Gene	Forward primer	Reverse primer	Product size (bp)
ANP	aac​cag​aga​gtg​agc​cga​ga	gtg​gtc​tag​cag​gtt​ctt​gaa​a	191
BNP	caa​tcc​acg​atg​cag​aag​ctg	ggc​gct​gtc​ttg​aga​cct​aa	132
Collagen Ⅰ	cca​act​gaa​cgt​gac​caa​aaa​cca	gaa​ggt​gct​ggg​tag​gga​agt​agg​c	345
Collagen Ⅲ	att​ctg​cca​ccc​tga​act​caa​gag​c	tcc​atg​tag​gca​atg​ctg​ttt​ttg​c	342
α-SMA	cta​ttc​ctt​cgt​gac​tac​t	atgctgttataggtggtt	255
GAPDH	tgg​cct​cca​agg​agt​aag​aaa​c	ggc​ctc​tct​ctt​gct​ctc​agt​atc	69

### Western Blot Analysis

The procedures for sample preparation and Western blot were conducted as described in our previous studies (Liu et al., 2014; Li et al., 2019a; Wang et al., 2019). Briefly, the RV tissues, H9c2 cells, or cardiac fibroblasts were homogenized with ice-cool lysis buffer (20 mM Tris, pH 7.5, 150 mM NaCl, and 1% Triton-X-100) with a protease and phosphatase inhibitor cocktail (Beyotime, Shanghai, China). And then, the protein concentration was detected according to the BCA assay kit instructions (Beyotime, Shanghai, China). Samples containing 30–40 μg of protein were subjected to 8 or 10% SDS-PAGE gel, and then they were transferred to polyvinylidene fluoride (PVDF) blotting membranes (G.E. Healthcare, Germany). The PVDF membranes were incubated with primary antibodies against collagen Ⅰ (Abcam, Cambridge, MA, USA), α-SMA (Cell Signaling Technology, Massachusetts, USA), p-JAK2 (Abcam, Cambridge, MA, USA), JAK2 (Beyotime, Shanghai, China), p-STAT3 (Signalway Antibody, Maryland, USA), STAT3 (Signalway Antibody, Maryland, USA), and α-tubulin (Santa Cruz, Texas, USA) followed by horseradish peroxidase (HRP) conjugated secondary antibody (Beyotime, Shanghai, China). The signals of Western blot bands were detected by BeyoECL Moon kit (Beyotime, Shanghai, China) through Molecular Imager ChemiDoc XRS System (Bio-Rad, Philadelphia, USA). Densitometric quantification was carried out by Image J (NIH, USA). The α-tubulin served as a loading control.

### Prediction of Potential Targets of Magnolol

SwissTargetPrediction (http://www.swisstargetprediction.ch/) was used to predict the potential targets of magnolol as described in the previous study (Daina et al., 2019). The structural information of magnolol was obtained from the PubChem database (https://pubchem.ncbi.nlm.nih.gov), and then the target-related information was obtained by using SwissTargetPrediction Database. Finally, the interaction between magnolol and potential targets was analyzed and mapped by Cytoscape (Shannon et al., 2003).

### Statistical Analysis

All quantitative data were presented as the means ± standard deviation (S.D.) and analyzed by using SPSS 20.0 software (SPSS, Chicago, United States). Dunnett’s test or the Student-Newman Keuls test was used for multiple comparisons after one-way analysis of variance (ANOVA). A probability level of *p* ≤ 0.05 was considered significant. ANOVA was used to compare the means among different groups.

## Results

### Magnolol Prevented Hypoxia-Induced PAH and RV Remodeling

**FIGURE 1:**
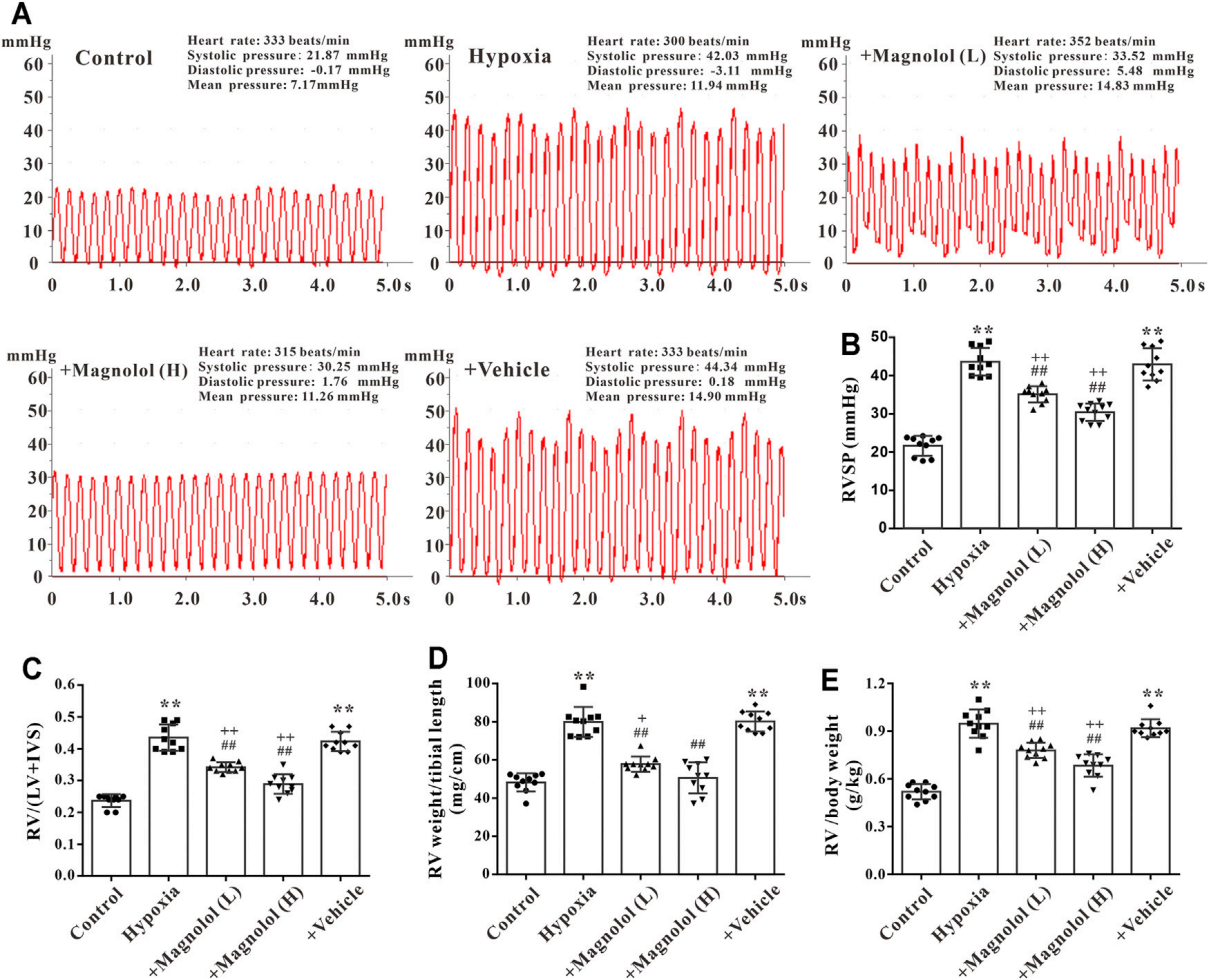
Magnolol prevented hypoxia-induced PAH and RV remodeling. **(A)** Representative images for right ventricular systolic pressure (RVSP) were measured by the right heart catheterization method. **(B)** The value of RVSP in each group. **(C)** The ratio of RV weight to left ventricular (LV) plus interventricular septum (IVS). **(D)** The ratio of RV weight to tibial length. **(E)** The ratio of RV weight to body weight. All values are presented as mean ± S.D. (*n* = 10 per group). **p < 0.01 vs. Control; ^+^ *p <* 0.05, ^++^ *p <* 0.01 vs. Control; ^##^ *p <* 0.01 vs. Vehicle.

A rat model of PAH was established after continuous exposure to hypoxia (10% O_2_) for 4 weeks. Compared with the control group, the right ventricular systolic pressure (RVSP) was significantly increased in the hypoxic group (Figures 1A,B); accompanied by the increases in the ratio of RV weight to left ventricle plus septum weight (RV/LV + IVS), RV weight to tibial length, and RV weight to body weight in the hypoxic group (Figures 1C–E), these phenomena were markedly attenuated by magnolol at both dosages (10 and 20 mg/kg). Interestingly, our study also found that magnolol can inhibit hypoxia-induced PAH vascular remodeling (Supplementary Figures S1), which is consistent with the results of Chang et al. (2018) *.*

### Magnolol Improved RV Function in the Hypoxic PAH Rats

**FIGURE 2:**
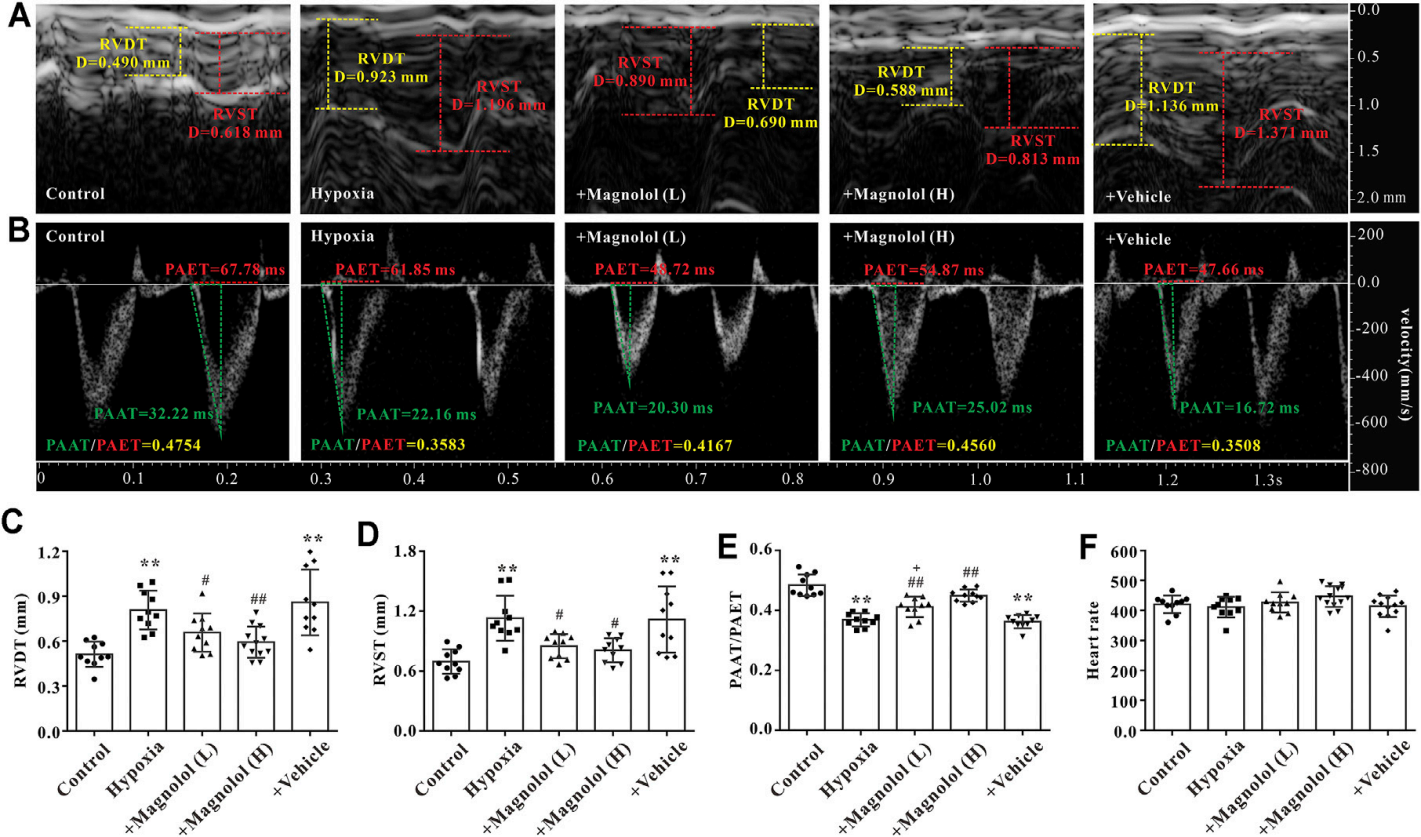
Magnolol improved RV dysfunction in hypoxia-induced PAH rats. **(A)** Representative images of right ventricular wall thickness in diastole and systole period. **(B)** Representative images of pulsed Doppler from pulmonary artery flow tract recorded in parasternal long axis, the pulmonary artery acceleration time (PAAT, green horizontal line), the pulmonary artery ejection time (PAET, red horizontal line). **(C)** The value of right ventricular wall thickness during the diastole period (RVDT). **(D)** The value of right ventricular wall thickness during the systole period (RVST). **(E)** The ratio of PAAT/PAET. **(F)** Heart rate. All values are presented as mean ± S.D. (*n* = 10 per group). **p < 0.01 vs. Control; ^+^ *p <* 0.05 vs. Control; ^#^ *p <* 0.05, ^##^ *p <* 0.01 vs. Vehicle.

Studies have shown that a sustained increase in pulmonary artery pressure can induce RV remodeling and eventually lead to right heart dysfunction and failure (Ciuclan et al., 2011). In this study, we found that the wall thickness of RV in the diastole and systole period was significantly increased in PAH rats (Figures 2A–D), accompanied by a decrease in the ratio of PAAT/PAET (Figure 2E); these phenomena were markedly attenuated by magnolol at both dosages (10 and 20 mg/kg). However, there was no significant difference in heart rate among all groups (Figure 2F).

### Magnolol Attenuated RV Hypertrophy in the Hypoxic PAH Rats

**FIGURE 3:**
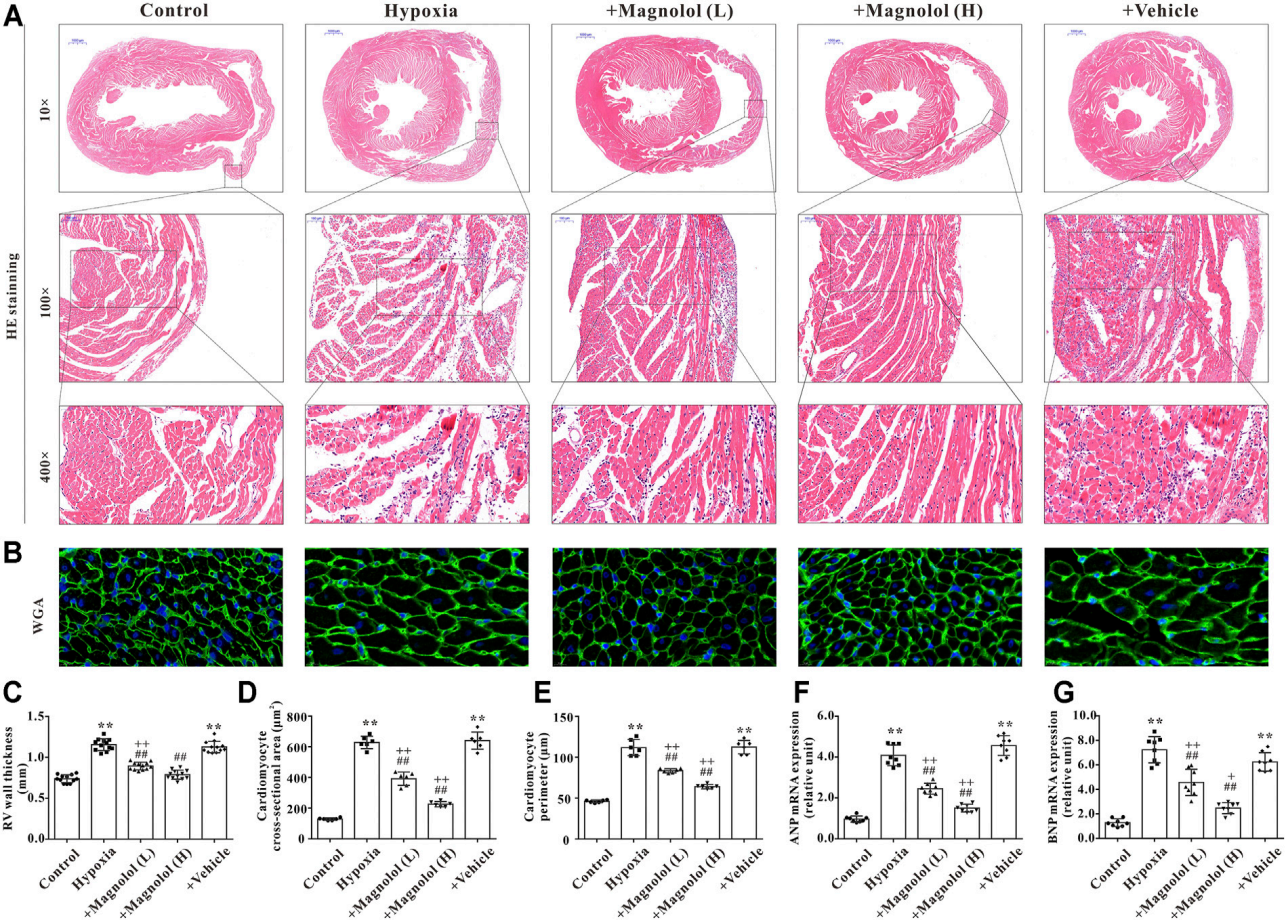
Magnolol attenuated RV hypertrophy in hypoxia-induced PAH rats. **(A)** Representative images of HE staining for heart tissues. **(B)** Representative images of WGA staining for RV tissues. **(C)** The right ventricular wall thickness (calculated from HE staining). **(D)** The cross-sectional area of cardiomyocytes (calculated from WGA staining). **(E)** The perimeter of cardiomyocytes (calculated from WGA staining). **(F)** The mRNA levels of ANP in RV tissues. **(G)** The mRNA levels of BNP in RV tissues. **p < 0.01 vs. Control; ^+^ *p <* 0.05, ^++^ *p <* 0.01 vs. Control; ^##^ *p <* 0.01 vs. Vehicle.

Our previous study has shown that myocardial hypertrophy is one of the fundamental causes of RV remodeling in rats with hypoxia-induced PAH (Li et al., 2019b). Therefore, the effect of magnolol on RV hypertrophy in the hypoxic PAH rats was evaluated by HE and WGA staining. Compared with the control group, the cross-sectional area and perimeter of cardiomyocytes were significantly increased in the hypoxia group (Figures 3A–E). Besides, the mRNA levels of atrial natriuretic peptide (ANP) and brain natriuretic peptide (BNP), two biomarkers of cardiac hypertrophy, were significantly up-regulated in RV tissues of PAH rats (Figures 3F,G); these phenomena were reversed in the presence of magnolol.

### Magnolol Attenuated RV Fibrosis in the Hypoxic PAH Rats

**FIGURE 4:**
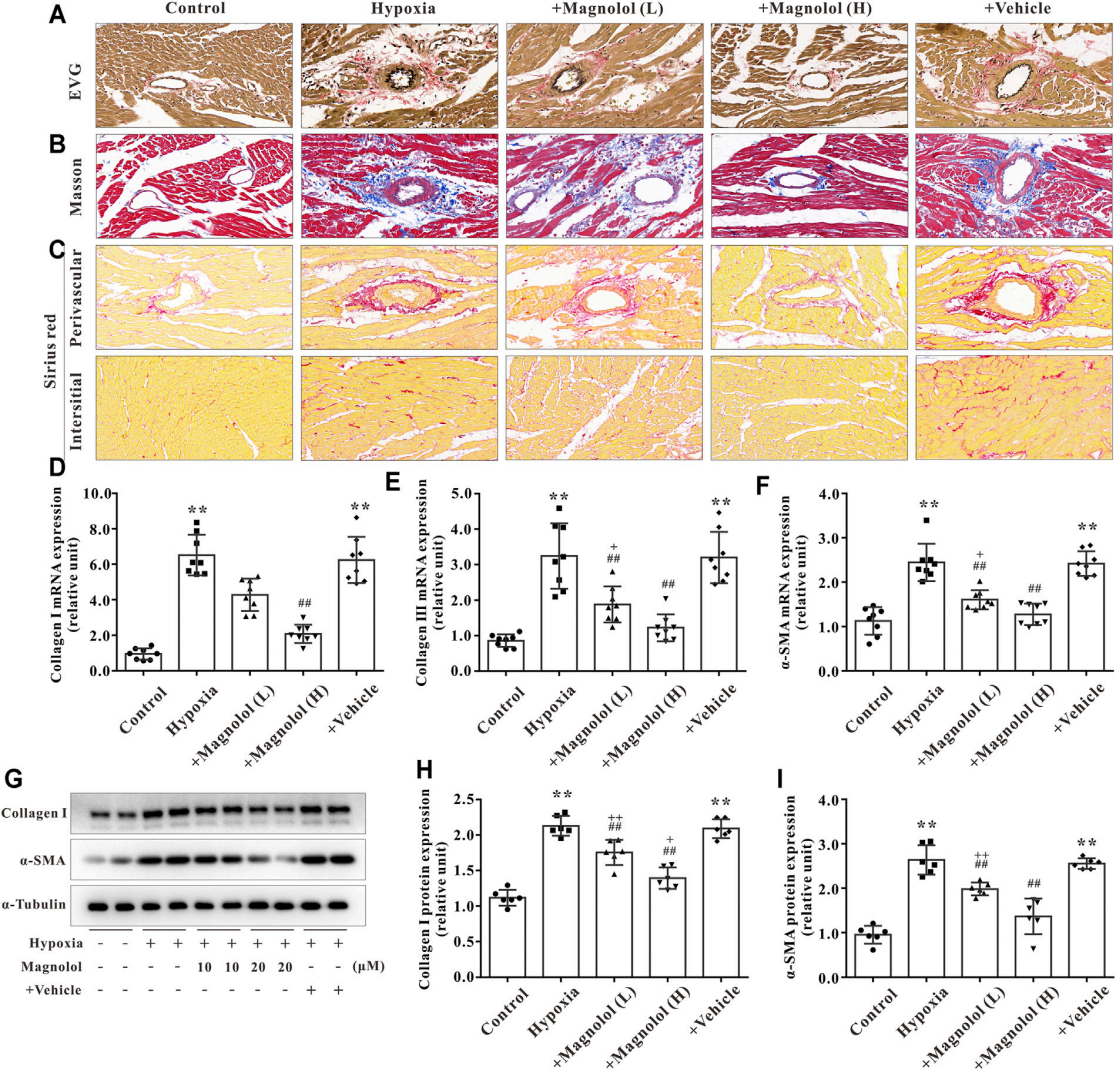
Magnolol attenuated RV fibrosis in hypoxia-induced PAH rats. **(A)** Representative images of EVG staining for RV tissues. The collagen fibers are red, and the elastic fibers are purple-black. **(B)** Representative images of Masson staining for RV tissues. The collagen fibers are blue. **(C)** Representative images of Sirius red staining for RV tissues. The collagen fibers are red, and the background is yellow. **(D)** The mRNA levels of Collagen Ⅰ in RV tissues. **(E)** The mRNA levels of Collagen Ⅲ in RV tissues. **(F)** The mRNA levels of α-SMA in RV tissues. **(G)** Representative images of Western blot results for Collagen Ⅰ, α-SMA, and α-tubulin. **(H, I)** The ratio of optical density between Collagen Ⅰ or α-SMA and α-tubulin. All values are presented as mean ± S.D. (*n* = 6–10 per group). **p < 0.01 vs Control; ^+^ *p <* 0.05, ^++^ *p <* 0.01 vs. Control; ^##^ *p <* 0.01 vs. Vehicle.

In addition to myocardial hypertrophy, myocardial fibrosis also plays a crucial role in the development of RV remodeling and failure. The formation of extracellular matrix (characterized by an excessive amount of collagen Ⅰ or Ⅲ) and the activation of myocardial fibroblasts into α-SMA are the main manifestations of myocardial fibrosis in PAH (Andersen et al., 2019). In this study, we observed that the collagen fibers in perivascular and interstitial RV tissue in hypoxia-induced PAH rats appeared to be increased, and those increased were inhibited by magnolol treatment (Figures 4A–C). To confirm these findings, we further found that the mRNA expression of collagen I, II, and a-SMA, as well as protein expression of collagen I and a-SMA, were significantly up-regulated in the RV tissue of hypoxic-treated rats; these targets were markedly attenuated by magnolol treatment (Figures 4D–I).

### Magnolol Blocked Hypoxia-Induced JAK2 and STAT3 Phosphorylation in RV Tissues

**FIGURE 5:**
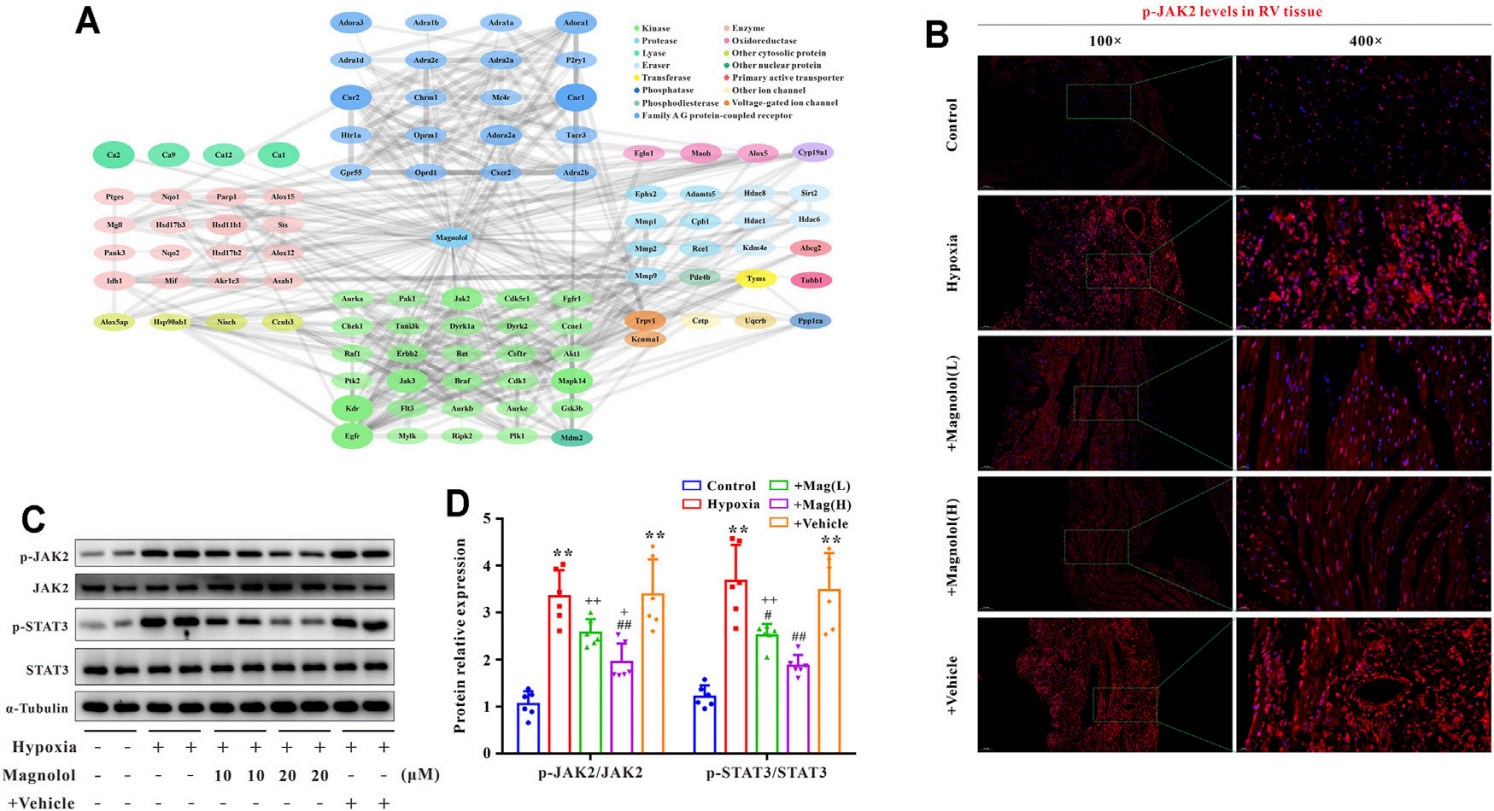
Magnolol blocks hypoxia-induced JAK2 and STAT3 phosphorylation in RV tissues. **(A)** Results from “SwissTargetPrediction” target prediction database. **(B)** Representative images of immunofluorescence staining for p-JAK2 in each group. **(C)** Representative images of Western blot results for p-JAK2, JAK2, p-STAT3, STAT3, and α-tubulin. **(D)** The ratio of optical density between p-JAK2/JAK2 or p-STAT3/STAT3. All values are presented as mean ± S.D. (*n* = 6 per group).**p < 0.01 vs. Control; ^+^ *p <* 0.05, ^++^ *p <* 0.01 vs. Control; ^#^ *p <* 0.05, ^##^ *p <* 0.01 vs. Vehicle.

To further confirm the potential target of magnolol in inhibiting RV remodeling in hypoxia-induced PAH, we used the “SwisstargetPrediction” target prediction database. We found that JAK2 was the potential target of magnolol (Figure 5A). A number of studies have reported that the activation of JAK2 could promote STAT3 phosphorylation and thus participate in the process of myocardial remodeling induced by angiotensin Ⅱ or PM_2.5_ (Ye et al., 2020; Xing et al., 2021). Immunofluorescence staining showed that the level of p-JAK2 was increased in the RV tissues of hypoxia-induced PAH rats (Figure 5B), suggesting that the protective effect of magnolol on the RV remodeling in PAH rats is related to target JAK2. This phenomenon was further confirmed by western blotting, as phosphorylation levels of JAK2 and STAT3 in RV tissue were evidently elevated in hypoxia-induced PAH rats, which were blocked in the presence of magnolol (Figures 5C,D). However, there were no changes in total JAK2 and STAT3 levels in all groups.

### Magnolol Attenuated Hypoxia-Induced H9c2 Cell Hypertrophy Through Inhibition of JAK2/STAT3 Signaling Pathway

**FIGURE 6:**
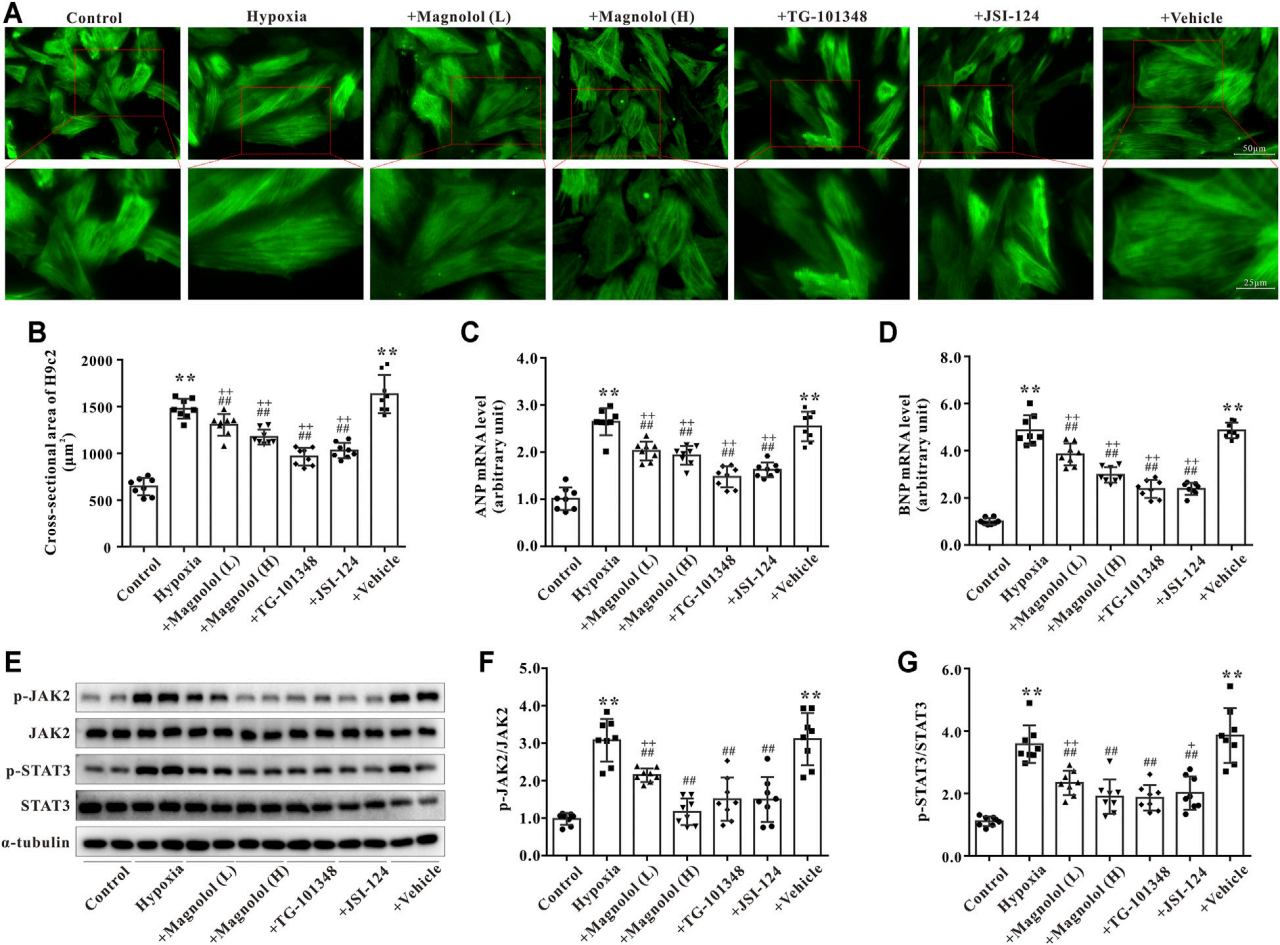
Magnolol attenuated hypoxia-induced cardiac hypertrophy of H9c2 through inhibition of JAK2/STAT3 signaling pathway. **(A)** Representative images for H9c2 cell morphology under the fluorescence microscope. The cells were incubated with primary antibodies against α-SMA followed by incubation with the secondary antibody of Alexa Fluor 488-labeled Goat Anti-Mouse IgG (green fluorescence). **(B)** The cross-sectional area of H9c2 cells. **(C)** The mRNA levels of ANP in H9c2 cells. **(D)** The mRNA levels of BNP in H9c2 cells. **(E)** Representative images of Western blot results for p-JAK2, JAK2, p-STAT3, STAT3, and α-tubulin in H9c2 cells. **(F)** The ratio of optical density between p-JAK2 and JAK2. **(G)** The ratio of optical density between p-STAT3 and STAT3. All values are presented as mean ± S.D. (*n* = 8 per group). **p < 0.01 vs. Control; ^+^ *p <* 0.05, ^++^ *p <* 0.01 vs. Control; ^##^ *p <* 0.01 vs. Vehicle.

As shown in Figures 6A,B, compared to the control group, the cross-sectional area of H9c2 was significantly increased in the hypoxia group, which was consistent with the results of our previous study (Li et al., 2019b). The hypoxia-induced H9c2 cell hypertrophy was inhibited by magnolol in a dose-dependent manner; TG-101348 or JSI-124 (JAK2 selective inhibitors) treatment showed a similar effect to magnolol, whereas the vehicle group had no such effect (Figures 6A,B). Consistent with the results *in vivo*, the mRNA expression of ANP and BNP in hypoxia-treated H9c2 cells were up-regulated; these increases were attenuated in the presence of magnolol, TG-101348, or JSI-124 (Figures 6C,D). The vehicle group had no such effects.

To further verify the potential mechanism of magnolol responsible for myocardial hypertrophy, the phosphorylation levels of JAK2 and STAT3 in hypoxia-treated H9c2 cells were observed. Consistent with the results *in vivo*, the phosphorylation levels of JAK2 and STAT3 in hypoxia-treated H9c2 were evidently elevated, which were attenuated in the presence of magnolol, TG-101348, or JSI-124 (Figures 6E–G). The vehicle group had no such effects. As shown in Supplementary Figure S2, under normoxic conditions, different doses of magnolol did not affect the phosphorylation levels of JAK2 and STAT3 in H9c2 cells.

### Magnolol Attenuated Hypoxia-Induced Fibrosis of Cardiac Fibroblasts Through Inhibition of the JAK2/STAT3 Signaling Pathway

**FIGURE 7:**
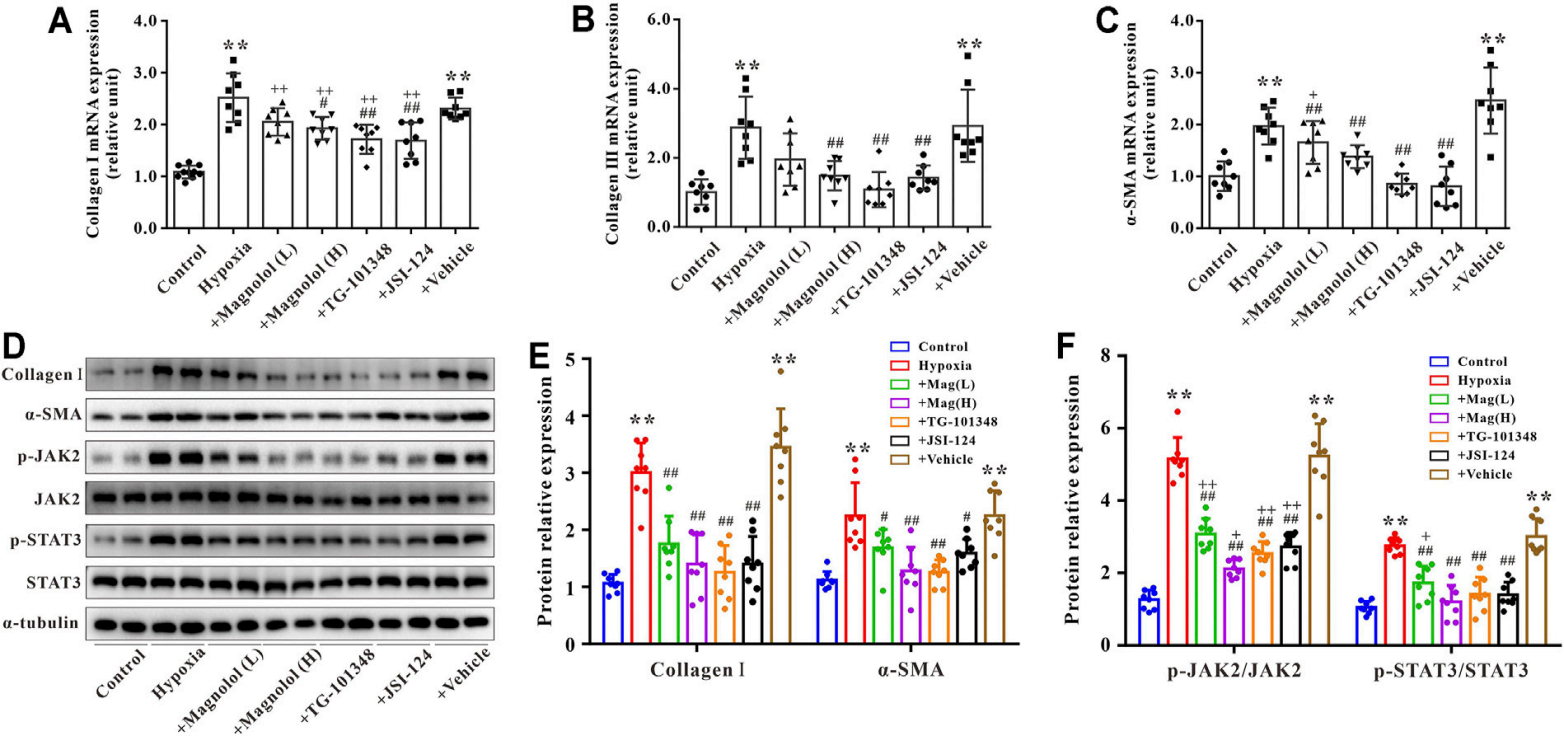
Magnolol attenuated hypoxia-induced myocardial fibrosis of cardiac fibroblasts through inhibition of JAK2/STAT3 signaling pathway. **(A)** The mRNA levels of Collagen Ⅰ in cardiac fibroblasts. **(B)** The mRNA levels of Collagen Ⅲ in cardiac fibroblasts. **(C)** The mRNA levels of α-SMA in cardiac fibroblasts. **(D)** Representative images of Western blot results for Collagen Ⅰ, α-SMA, p-JAK2, JAK2, p-STAT3, STAT3, and α-tubulin in cardiac fibroblasts. **(E)** The ratio of optical density between Collagen Ⅰ or α-SMA and α-tubulin. **(F)** The ratio of optical density between p-JAK2/JAK2 or p-STAT3/STAT3. All values are presented as mean ± S.D. (*n* = 8–10 per group). **p < 0.01 vs. Control; ^+^ *p <* 0.05, ^++^ *p <* 0.01 vs. Control; ^#^ *p <* 0.05, ^##^ *p <* 0.01 vs. Vehicle.

Primary cardiac fibroblasts were cultured under hypoxic conditions for 24 h to establish an *in vitro* model of myocardial fibrosis. As shown in Figure 7, compared to the control group, the mRNA expressions of collagen Ⅰ, Ⅲ, and α-SMA, as well as the protein expressions of collagen Ⅰ and α-SMA in the hypoxia group were significantly up-regulated; these increases were attenuated by magnolol in a dose-dependent manner. Similarly, TG-101348 or JSI-124, the specific inhibitor of JAK2, could also mitigate the myocardial fibrosis induced by hypoxia, but the vehicle has no such effects (Figures 7A–E). Western blotting results showed that magnolol could inhibit the phosphorylation of JAK2 and STAT3 induced by hypoxia, similar to that of TG-101348 or JSI-124 did (Figures 7D,F). The vehicle had no such effects.

## Discussion

In this study, we explored the effects of magnolol on RV hypertrophy and fibrosis in hypoxia-induced PAH rats and the underlying mechanisms. The results from animal experiments demonstrated that administration of magnolol significantly prevented RV remodeling and dysfunction in PAH rats, accompanied by a decrease in the phosphorylation levels of JAK2 and STAT3. In hypoxia-treated H9c2 or cardiac fibroblasts, the cross-sectional area and mRNA levels of ANP/BNP in H9c2 were significantly increased, the expressions of collagen Ⅰ, Ⅲ, and α-SMA in cardiac fibroblasts were also elevated, concomitant with an increase of phosphorylation levels of JAK2 and STAT3; these phenomena were blocked in the presence of magnolol. To the best of our knowledge, this is the first study to provide evidence that magnolol prevents hypoxia-induced RV hypertrophy and fibrosis through inhibition of the JAK2/STAT3 pathway.

PAH is a progressive disease that can affect the normal function of pulmonary vessels and the heart (Vonk-Noordegraaf et al., 2013; Frost et al., 2019). The continuous increase of pulmonary vascular resistance and pressure during PAH leads to an increase in RV afterload, which in turn induces RV remodeling. The constant expansion of the RV eventually causes right heart function damage and even right heart failure (Vonk Noordegraaf et al., 2017). In this paper, we observed a significant increase in the RVSP accompanied by an elevation in the index of RV/(LV + IVS), RV weight/tibial length, and RV weight/body weight in rats exposed to hypoxia for 4 weeks. In addition, our study also found that the RV function of rats was significantly impaired after 4 weeks of hypoxic treatment, manifesting as a significant increase in the wall thickness of the right ventricle during diastole and systole (RVDT and RVST), and a considerable reduction in the ratio of PAAT/PAET. These results indicate that the hypoxia-induced PAH rat model was successfully established.

It is well recognized that hypoxia-induced myocardial hypertrophy is the main pathological feature of RV remodeling in PAH (Zhu et al., 2017; Smith et al., 2020). Cardiac hypertrophy includes physiological and pathological hypertrophy, both manifest as the expansion of a single cardiomyocyte, but the characteristics of them are different (Nakamura and Sadoshima, 2018). Hypertrophy under physiological conditions is mainly characterized by an increase in the mass, length, and width of individual cardiomyocyte to increase myocardial contractility and maintain normal cardiac output. The above process is reversible and will not develop into heart failure. Different from physiological hypertrophy, cardiomyocytes gradually develop from the initial compensatory hypertrophy to decompensated hypertrophy under pathological conditions, which is mainly characterized by myocardial contractile dysfunction and heart failure. During this process, the expression of ANP and BNP, which is commonly regarded as a marker of heart failure, is significantly increased, accompanied by interstitial and perivascular fibrosis and myofibroblast activation (Nakamura and Sadoshima, 2018). In this study, by using HE and WGA staining, we found that the wall thickness of the RV, the cross-sectional area, and perimeter of individual cardiomyocyte significantly increased in rats exposed to hypoxia for 4 weeks, accompanied by an increase in the mRNA expressions of ANP and BNP in the RV tissue, confirmed the role of myocardial hypertrophy in RV remodeling in hypoxic PAH.

In addition to myocardial hypertrophy, myocardial fibrosis is also one of the critical factors leading to RV remodeling during PAH (Simpson and Hassoun, 2019; Tian et al., 2020). As mentioned above, the increase in RV afterload during PAH also leads to the activation of myofibroblasts, leading to myocardial fibrosis and an increase of extracellular matrix. In this study, we observed an elevation in the production of collagen fibers in interstitial and perivascular of RV, concomitant with a significant increase in the levels of myocardial fibrosis markers such as collagen Ⅰ, Ⅲ, and α-SMA, suggesting that myocardial fibrosis also plays a pivotal role in the process of RV remodeling.

Janus kinase 2 (JAK2) is an essential member of the non-receptor tyrosine kinase family, promoting the phosphorylation of signal transducer of activators of transcription 3 (STAT3). The activated STAT3 can enter the nucleus and participate in cell growth, differentiation, and apoptosis by regulating the expression of downstream target genes (Montero et al., 2021). Growing evidence has shown that JAK2/STAT3 pathway plays a vital role in the pathogenesis of PAH. Yerabolu et al. found that, compared with healthy individuals, the phosphorylation level of JAK2 in pulmonary artery smooth muscle cells derived from PAH patients was significantly increased. Using monocrotaline or hypoxia-induced PAH animal models, Yerabolu et al. demonstrated that ruxolitinib (JAK2 inhibitor) could inhibit PAH vascular remodeling and improve RV function (Yerabolu et al., 2021). Other studies reported that administration of JAK2 specific inhibitors, such as TG-101344 (also named Fedratinib) (Zhang et al., 2020) or JSI-124 (Milara et al., 2018), can delay the progression of PAH by inhibiting the activation of the JAK2/STAT3 pathway. Collectively, these studies have confirmed that the JAK2/STAT3 pathway plays a pivotal role in PAH vascular remodeling, but its role in RV remodeling has not been fully elucidated. The latest research found that the JAK2/STAT3 pathway participates in the process of AngⅡ-induced myocardial remodeling by promoting myocardial hypertrophy and fibrosis (Ye et al., 2020). In this study, we found that the phosphorylation levels of JAK2 and STAT3 were evidently elevated in the RV of hypoxic PAH rats, and the same results can be observed in hypoxia-treated H9c2 cells or cardiac fibroblasts. These results indicate that the JAK2/STAT3 pathway contributes to the process of hypoxia-induced cardiac hypertrophy and fibrosis. Therefore, the intervention of the JAK2/STAT3 pathway with specific drugs may provide a new strategy for preventing RV remodeling in PAH.

Magnolol is one of the foremost effective ingredients of traditional Chinese medicine *Magnolia Officinalis*, which has received widespread attention due to its anti-inflammatory (Zhou et al., 2019) and anti-tumor (Chen et al., 2021a) effects. A recent study reported that magnolol could ameliorate the vascular remodeling in monocrotaline-induced PAH rats (Chang et al., 2018). However, the impact of magnolol on RV remodeling in PAH remains unclear. In addition, Chen et al*.* confirmed that magnolol could inhibit cardiac fibroblasts’ proliferation and collagen synthesis (Chen et al., 2021b). Expectedly, in the present study, we found that magnolol had a preventive effect on RV remodeling in hypoxia-induced PAH rats by inhibiting abnormal myocardial hypertrophy and excessive collagen fiber production. However, it is noteworthy that our current results can only prove that magnolol has a preventive effect on RV remodeling in PAH. Although magnolol may likely have a therapeutic effect on PAH, more studies are needed before drawing a firm conclusion.

To further confirm the possible mechanisms for magnolol against myocardial hypertrophy and fibrosis, the bioinformatics analysis was used to predict the potential targets for magnolol. Actually, nearly 100 potential targets have been identified (including kinases, oxidoreductases, and proteases). Among them, JAK2 and STAT3 have been attracted our attention because the JAK2/STAT3 pathway is closely related to myocardial hypertrophy and fibrosis (Ye et al., 2020). In addition, accumulating evidence indicates that magnolol has a regulatory effect on the activity of STAT3 (Chen et al., 2006; Peng et al., 2021). Based on the reports and bioinformatics prediction, we hypothesize that magnolol prevents RV remodeling in hypoxia-induced PAH rats via suppressing the JAK2/STAT3 pathway. We thus examined the correlation between Magnolol and JAK2/STAT3 pathway. The results from the present study revealed that the levels of p-JAK2 and p-STAT3 in RV tissues were apparently elevated in the hypoxia-treated rats, which were reversed in the presence of magnolol. According to the results of bioinformatics analysis, the potential targets of magnolol also include kinases such as Raf1 or Akt1, which play an essential role in cardiac remodeling. Here, we could not rule out the role of Raf1 and/or Akt1 in the preventive effect of magnolol on RV remodeling.

To further confirm our findings *in vivo*, we performed cell experiments in H9c2 or primary cultured cardiac fibroblasts with Magnolol, TG-101348, and JSI-124. Here, TG-101348 or JSI-124 served as the positive control for JAK2 inhibitors, both of which have been proven to delay the development of PAH (Milara et al., 2018; Zhang et al., 2020). Consistent with the findings *in vivo*, H9c2 or cardiac fibroblasts displayed cell hypertrophy and fibrosis respectively under hypoxic conditions, accompanied by an elevation in p-JAK2 and p-STAT3 levels; these phenomena were attenuated in the presence of magnolol, TG-101348, or JSI-124.

There are two major limitations that need to be acknowledged and addressed regarding the present study. Firstly, as we mentioned above, pulmonary vascular remodeling is the most important pathological feature of PAH, which can induce an increase in RV afterload and ultimately lead to RV remodeling. Consistent with the results of Chang et al. (2018), we also found that magnolol has the effect of inhibiting pulmonary vascular remodeling. Although we have confirmed *in vitro* that magnolol has a protective effect on hypoxia-induced myocardial hypertrophy and fibrosis, we cannot rule out the beneficial effects of magnolol on RV remodeling and function are due to, at least in part, its indirect effect on inhibition of pulmonary vascular remodeling. Secondly, our existing results can only confirm that magnolol has a preventive effect on hypoxia-induced PAH right ventricular remodeling. Further studies are needed to verify the therapeutic effect of magnolol on RV hypertrophy and fibrosis.

## Conclusion

In summary, the results presented here demonstrated for the first time that magnolol can prevent RV hypertrophy and fibrosis in hypoxia-induced PAH rats through a mechanism involving suppression of the JAK2/STAT3 pathway. Therefore, magnolol may have the potential to treat PAH.

## Data Availability

The original contributions presented in the study are included in the article/Supplementary Material, further inquiries can be directed to the corresponding author.

## Supplementary Material

Supplementary Figure S1 Magnolol attenuated hypoxia-induced pulmonary vascular remodeling. **(A)** Representative images of HE staining for lung tissues. **(B)** The ratio of wall thickness to total vessel diameter. **(C)** The ratio of wall area to total vessel area. All values are presented as mean ± S.D. (*n* = 10 per group). ***p <* 0.01 vs. Control; ^+^ *p <* 0.05 vs. Control; ^##^ *p <* 0.01 vs. Vehicle.

Supplementary Figure S2 Effect of magnolol on the phosphorylation of JAK2 and STAT3 in normal H9c2 cells. **(A)** Representative images of Western blot results for p-JAK2, JAK2, p-STAT3, STAT3, and α-tubulin in H9c2 cells. **(B–C)** The ratio of optical density between p-JAK2/JAK2 or p-STAT3/STAT3. *n* = 3 per group.
