# Supplementary figures and images for: Magnolol Attenuates Right Ventricular Hypertrophy and Fibrosis in Hypoxia-Induced Pulmonary Arterial Hypertensive Rats Through Inhibition of the JAK2/STAT3 Signaling Pathway
Source: Front Pharmacol. 2021 Oct 26;12:755077. doi: 10.3389/fphar.2021.755077 (PMC8576411; doi:10.3389/fphar.2021.755077)

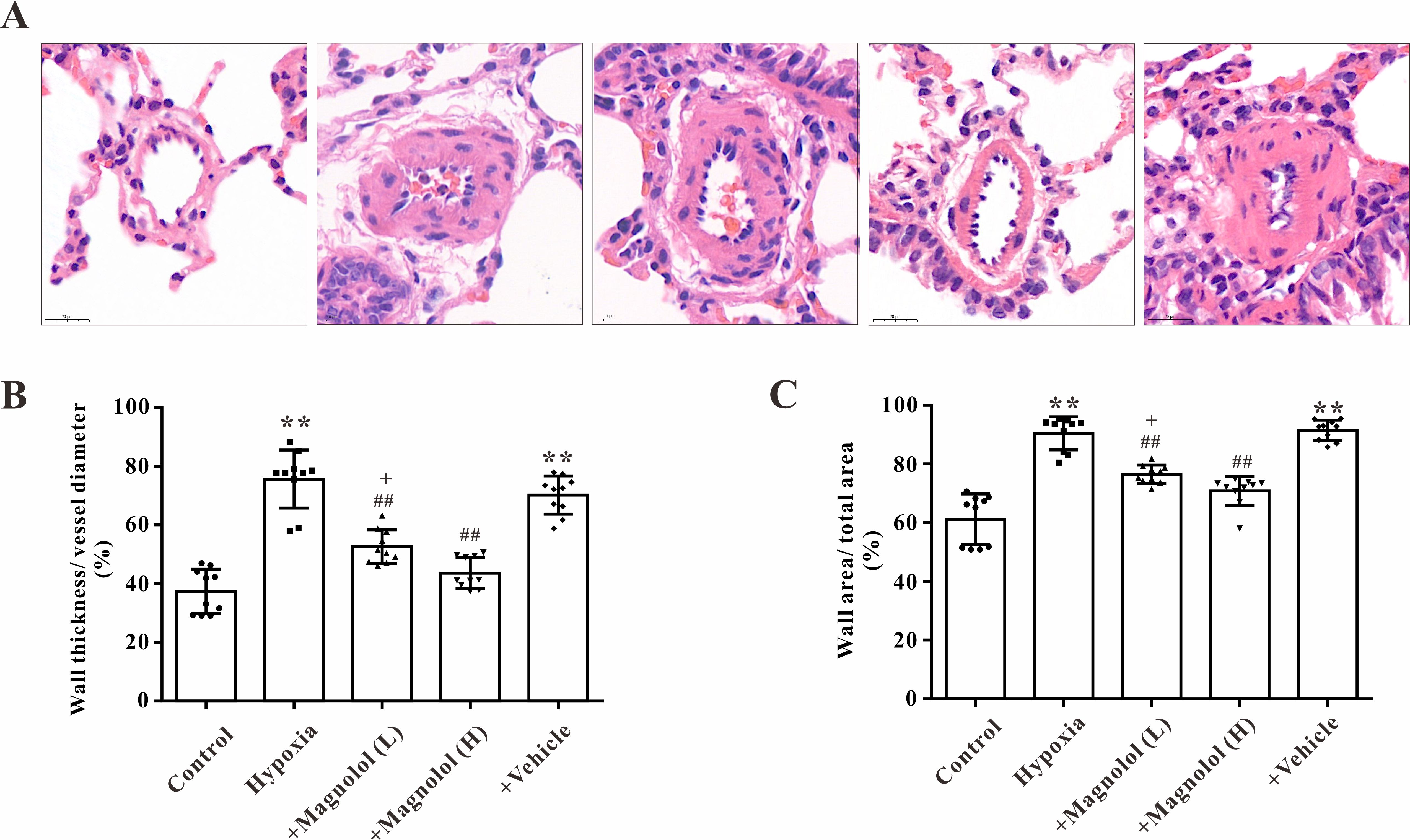

Supplement: Supplementary file 1 [file Image1.JPEG]

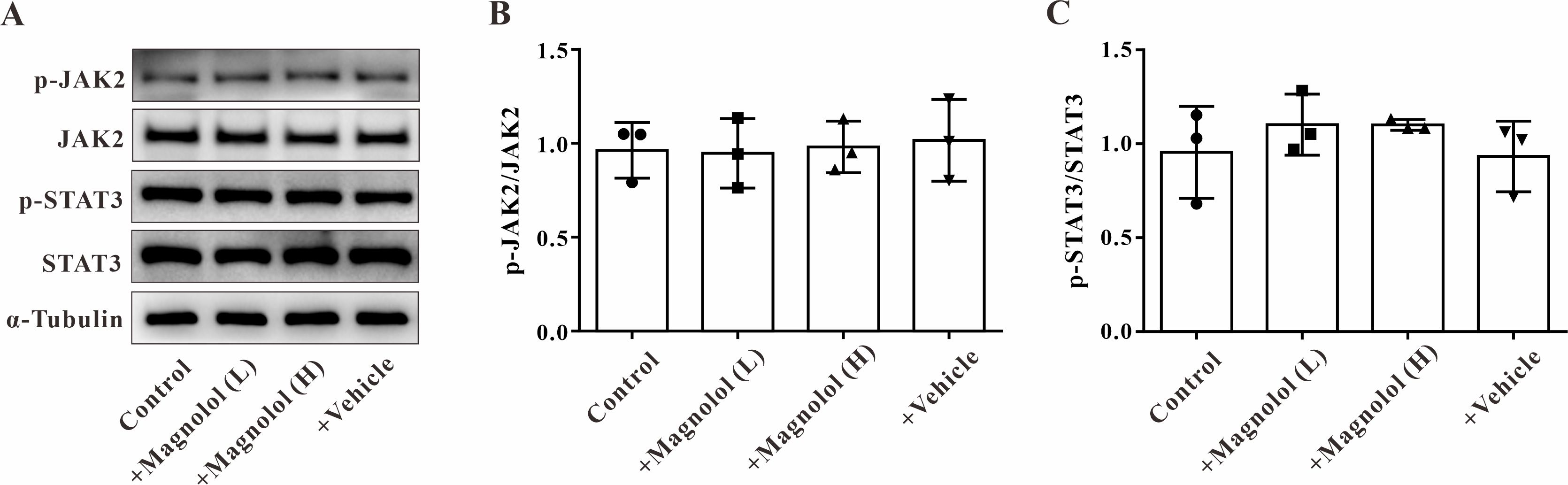

Supplement: Supplementary file 2 [file Image2.JPEG]
